# Socioeconomic Position and Adolescent Trajectories in Smoking, Drinking, and Psychiatric Distress

**DOI:** 10.1016/j.jadohealth.2013.02.023

**Published:** 2013-08

**Authors:** Michael J. Green, Alastair H. Leyland, Helen Sweeting, Michaela Benzeval

**Affiliations:** MRC/CSO Social and Public Health Sciences Unit, Glasgow, Scotland

**Keywords:** Smoking, Alcohol, Psychiatric distress, Socioeconomic position, Adolescence, Development, Latent class analysis, Longitudinal

## Abstract

**Purpose:**

Smoking, drinking, and psychiatric distress are inter-related and may also be associated with socioeconomic position (SEP). This paper investigates the role of SEP in adolescent development across all three of these outcomes.

**Methods:**

Data were self-reported by adolescents in the Twenty-07 Study (N = 1,515) at ages 15, 17, and 18 years. Latent class analysis was used to identify homogeneous subgroups of adolescents with distinct developmental patterns. Associations between developmental patterns and a range of socioeconomic indicators were then tested.

**Results:**

Five classes were identified. A *Low Risk* class had low levels for all outcomes. A *High Distress* class had persistently high levels of distress, but was otherwise similar to the *Low Risk* group. A *High Drinking* class drank alcohol earlier and more heavily but also had higher levels of distress than the *Low Risk* group. Smokers were grouped in two classes, *Early Smokers* and *Late Smokers*, and both also had raised levels of drinking and distress. *Early Smokers* tended to begin earlier and smoke more heavily than *Late Smokers*. Relative to the *Low Risk* class, adolescents in a disadvantaged SEP were more likely to be *Early Smokers* and somewhat less likely to be in the *High Drinking* class. SEP was not consistently associated with membership in the *High Distress* or *Late Smokers* classes.

**Conclusions:**

Associations with SEP are evident in opposing directions or absent depending on the combination and timing of outcomes, suggesting that a disadvantaged SEP is not a simple common cause for all three outcomes.

**Implications and Contribution**

A disadvantaged socioeconomic position is specifically associated with a developmental pattern where smoking begins early and higher levels of drinking and distress follow. Outside of this pattern, drinking and distress appear somewhat more common among more affluent adolescents. Such opposing processes are only apparent when examining these outcomes in combination.

Smoking and excessive alcohol consumption (hereafter referred to as drinking) are related to psychiatric distress (or symptoms of anxiety and depression) in both adolescent and adult populations. These behaviors and symptoms usually begin in adolescence and continue into adulthood [1,2]. Prospective data from adolescents suggest reciprocal relationships with distress leading to smoking and drinking and vice versa [3–5]. Alcohol and tobacco may be used as forms of “self-medication” to manage psychiatric distress, and/or the use of these substances may pre-dispose a person to developing psychiatric symptoms, either through the physiological effects of substance use, or via the disruption of social relationships [6–8]. All three outcomes represent important public health problems: all are associated with mortality [9–11], smoking and drinking carry risks for chronic disease [12,13], and psychiatric distress can be disabling [14], so it is important to understand their development. However, considering the prospective associations among these outcomes, there could be significant benefits to examining development holistically across all three. This may help provide insights as to when secondary prevention efforts might be most effective, and improve understanding of etiology [7], because the processes that lead to one of these outcomes occurring in isolation may be different from those processes that lead to them occurring together [15].

One potentially important etiological factor is a person's socioeconomic position (SEP), which could influence each outcome via the stratification of social and economic resources or stressors. If SEP is a common cause then this may explain the associations among these outcomes, though an etiological role of SEP does not exclude further pathways linking the outcomes to each other such as those suggested above. Although adolescents in a disadvantaged SEP are more likely to smoke [16] and experience depressed mood [17], studies on SEP and adolescent drinking vary, showing associations in either direction or no relationship at all [16]. However, these studies have tended to treat each outcome individually, without accounting for the relationships among them. The role of SEP may be clearer if these outcomes are examined together.

This paper aims to identify the most common patterns of adolescent development in smoking, drinking, and psychiatric distress and see whether a disadvantaged SEP is associated with all patterns of increased health risk, or only with specific developmental patterns. Latent class analysis [18] is employed to identify distinct groups of adolescents with similar patterns of development, and then relate membership in those groups to SEP. SEP is commonly measured using a variety of indicators, but each may emphasize particular characteristics [19]. A range of SEP measures are employed to assess whether the associations are robust to measurement differences. Gender is also adjusted for as an important adolescent correlate of these outcomes [20,21].

## Methods

### Sample

Data are from the Twenty-07 Study based in and around Glasgow in the West of Scotland [22]. People in three age cohorts have been followed for 20 years. This paper involves the youngest cohort, who had a baseline response rate of 85%. Baseline interviews with the respondents and their parents were conducted in 1987 (n = 1,515), a postal survey was conducted approximately 1 year later (n = 1,250), and further follow-up interviews took place in 1990 (n = 1,343). The mean age of the respondents was 15.7, 17.1, and 18.6 years respectively at each of these time-points. Ethical approval was obtained for each wave of data collection from the National Health Service (NHS) and/or Glasgow University Ethics Committees. Written parental consent for respondent's participation was obtained at the start of the baseline interview and from the respondents themselves at the follow-up interview. Regarding the postal survey, consent was indicated by return of the questionnaire. Baseline respondents were representative of the general population of the sampled area [23].

### Measures

#### Outcomes

Respondents self-reported each outcome at each measurement point. Regarding smoking and drinking, respondents were asked about their current status and then for further detail on quantity/frequency if they were current smokers/drinkers. For smokers the number of cigarettes smoked daily was obtained (dividing by 7 where respondents had reported weekly amounts). At baseline drinkers reported the frequency of their drinking, while in the two follow-up surveys they reported their drinking in detail over the past 7 days. Psychiatric distress was assessed using the 12-item General Health Questionnaire (GHQ-12) [24].

A four-category measure was constructed for each outcome to cover the range from no use or no symptoms to heavy use or severe symptom levels. Smoking was categorized at each survey into: not currently smoking, smoking fewer than 1-a-day, smoking regularly (1-a-day or more), and smoking heavily (10-a-day or more). At baseline, drinking was categorized according to the available information into: not currently drinking, drinking less than monthly, monthly drinking, and weekly drinking. At the two later surveys, drinking was categorized into: not currently drinking, drinking less than weekly, weekly drinking within UK recommended limits in the past week (14 units for females, 21 for males) [25], and weekly drinking exceeding recommended limits in past week. Psychiatric distress was categorized using GHQ-12 scores into: no (0), mild (1–2), medium (3–4), and severe symptoms (5+). Across all measures, for convenience, the four categories will be referred to as: none, low, medium, and high.

### Covariates

Gender was coded 1 for females, 0 for males. All SEP indicators came from the parental interview at baseline, and were based on parental or household characteristics. They are viewed as representing the SEP of the households in which the adolescents were being raised and are thus considered conceptually as antecedent to the outcomes. Household social class was coded according to the UK Registrar General's 1980 classification [26], using the higher status occupation from couple parents, dichotomized into manual and nonmanual categories. Lone parenthood differentiated between respondents who had a single parent and those whose parents were married or co-habiting, and is viewed as a marker for socioeconomic disadvantage. Housing tenure dichotomized those in owned or mortgaged accommodation and those in rented or other types of accommodation. Parental education (taking the higher value from couples) separated those with and without education beyond the age of 16 years. Parental employment status was coded in three categories for the most economically active parent in the household: full-time, part-time, or not employed. Parents reported whether their weekly household income after tax was less than £50, £50–99, £100–149, £150–199, £200–249, £250–299, £300–349, £350–399, £400–449, £450–499 or greater than £500. The mid-point of the chosen band was equivalized for household composition [27], and the equivalized household income variable was split into tertiles. Area deprivation was based on Carstairs scores for baseline postcode sectors (average population = 5,000) derived from the closest Census information (1991) [28]. Carstairs scores provide an index of deprivation based on proportions of: households in the area that are overcrowded; heads of household in the area who are in social classes IV and V; male heads of household in the area who are unemployed; and households in the area that do not have access to a car. Scores are commonly split into seven groups referred to as deprivation categories. These were further grouped into: least deprived (1–2); middling (3–5); and most deprived (6–7).

### Analysis

Analyses were performed using Mplus version 7 [29] and models were estimated using maximum likelihood under the missing-at-random (MAR) assumption (i.e., that missingness is random given the other variables in the model) [30]. The analysis proceeded in two stages. First, latent class analysis [18] was used to identify patterns of development across the three outcomes over the three measurement points. Latent classes represent the most common and distinct developmental patterns, with each latent class having a profile of response probabilities detailing the likelihood of each outcome at each measurement. The number of latent classes was determined by estimating a series of latent class models each with an incrementally greater number of classes and then comparing these models on the basis of various model-fit statistics. Models with greater interpretative value were chosen where fit-statistics did not point to a single optimal model (see Appendix 1 in the online edition of this article for details). Two respondents were excluded at this stage because they had missing data on all of the outcome variables at all measurements (n = 1,513). Males and females could potentially have exhibited substantively different developmental patterns, so this stage of modeling was also carried out on males and females separately. Similar groupings were identified but at different frequencies (results not shown). Including gender as a predictor of class membership in the next stage of modeling was therefore deemed adequate for capturing gender differences in developmental patterns.

Associations between SEP and latent class membership were examined in the second stage of modeling. Latent class analysis provides for each respondent the probability of being in each class given their observed responses. A common practice is to assign respondents to the class where they have the highest probability of membership and then treat these modal class assignments as if observed in further analyses. This, however, does not take account of the uncertainty in class membership and therefore tends to underestimate the magnitude of associations with covariates [31]. In order to account for such uncertainty, this paper uses the 3-step modal maximum-likelihood procedure described by Vermunt [31]. This procedure performs well at identifying true relationships between latent class membership and covariates in simulation studies [31,32]. Each SEP indicator was included in a separate multinomial regression of latent class membership. All models were adjusted for gender, and interactions between gender and SEP indicators were examined. This stage of modeling used only those respondents with full data on all SEP covariates (n = 1,383), but for consistency the response probability parameters of the latent class model were fixed to those values identified in the previous stage. Modal class assignments for those who were excluded because of missing covariate information did not differ significantly from the class assignments of those who were included (chi-square; *p* = .12). The analysis was also performed using modal class assignments with similar findings (see Appendix 2 in the online edition of this article for details), except that the odds ratios (ORs) for modal assignments tended to be closer to unity and have smaller standard errors than those from the Vermunt 3-step method.

## Results

Table 1 shows descriptive statistics for the covariates, and the proportion of those with these baseline characteristics at the two follow-ups. Drop-out was somewhat greater among males and those in a disadvantaged SEP, but these differences were not large.

Table 2 shows the prevalence of different levels of smoking, drinking, and psychiatric distress over the three measurement points. For all three outcomes, changes between ages 15 and 18 years mainly reflected shifts toward higher prevalence and heavier consumption or more severe symptoms.

**Figure 1:**
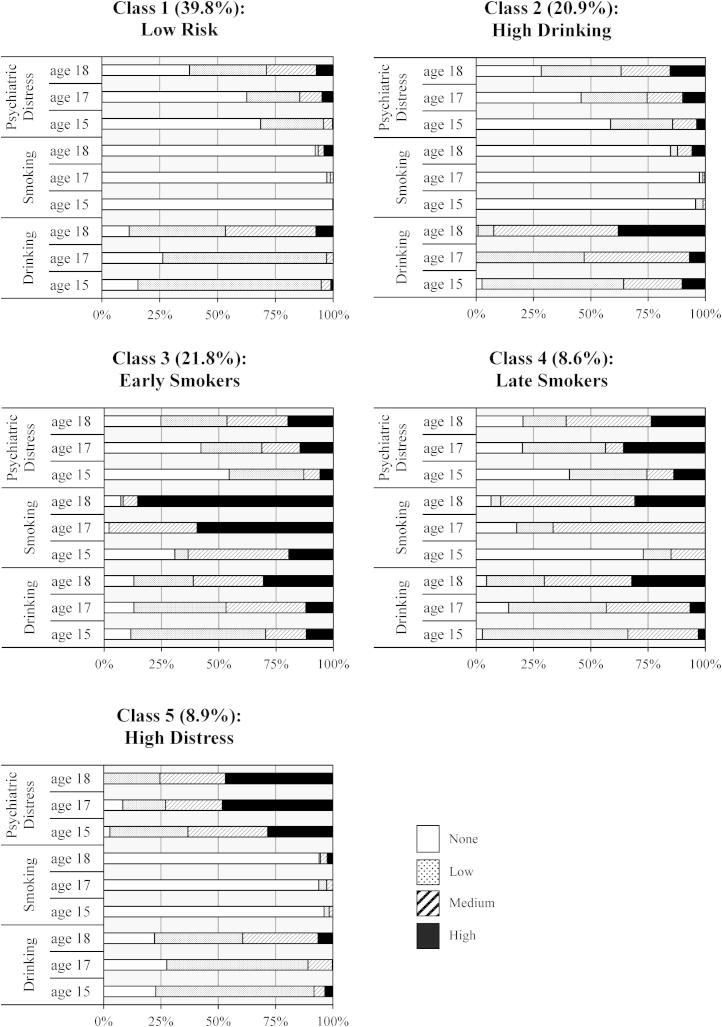
Latent class response probability profiles.

A model with five latent classes was selected as the optimal description of the developmental profiles within the smoking, drinking, and psychiatric distress data (see Appendix 1 online). Figure 1 displays the proportions at each level of smoking, drinking, and psychiatric distress within each of the five latent classes. Class 1 had the healthiest pattern of responses: they had the lowest levels of psychiatric distress, which increased modestly with age; mainly low drinking, with some progressing to medium drinking by age 18; and very little smoking. We label this group *Low Risk*. Class 2 is labeled *High Drinking* because they started drinking earlier and many were drinking heavily by age 18. This group contained very few smokers but had higher distress levels than in the *Low Risk* class. Class 3 is labeled *Early Smokers* because there were many medium smokers at age 15 years with the majority smoking 10-a-day or more by age 17. *Early Smokers* also had greater increases with age in both distress and earlier and heavier involvement with drinking than those in the *Low Risk* class. Class 4 had relatively high levels of distress and a similar drinking pattern to that of the *Early Smokers*, but tended to take up smoking later and to smoke less than 10-a-day, so they are labeled *Late Smokers*. In this group the three problems appeared to develop more or less concurrently, whereas smoking tended to precede the development of drinking and distress problems among the *Early Smokers*. Finally, Class 5 is labeled *High Distress* because they had persistent and severe psychiatric symptoms across the three surveys, but were otherwise similar to the *Low Risk* class, with low levels of smoking and drinking. The estimated proportions in each class were as follows: *Low Risk* (39.8%); *High Drinking* (20.9%); *Early Smokers* (21.8%); *Late Smokers* (8.6%); and *High Distress* (8.9%).

Table 3 shows the odds ratios (OR) for membership in each class relative to the *Low Risk* class, for gender and SEP. Females were more likely to be in the *High Distress* and *Late Smokers* classes and less likely to be in the *High Drinking* class than males. Four of the seven indicators of a disadvantaged SEP were associated with lower odds of membership in the *High Drinking* class (*p* < .05 for housing tenure and area deprivation; *p* ≤ .1 for social class and income). Associations between most of the other indicators of a disadvantaged SEP and being in the *High Drinking* class showed trends in the same direction, but did not reach statistical significance. There was also a gender interaction (not shown) such that females with unemployed parents were less likely to be in this group (*p* < .05). All indicators of a disadvantaged SEP (except those for area deprivation) were associated with increased odds of being *Early Smokers*. In contrast, all SEP indicators showed a trend toward lower odds of being *Late Smokers* for those in a disadvantaged SEP, but this only reached statistical significance for area deprivation. For the *High Distress* class, there were significant associations with SEP in opposite directions for different measures: adolescents from lone parent families were more likely to be in this group and those from more deprived areas were less likely to be in this group. Those whose parents had less education were also somewhat less likely to be in this group (*p* < .1). However, most of the SEP indicators did not show significant associations with membership in this class. No other interactions between gender and SEP were observed (*p* < .05).

## Discussion

Distinct patterns of adolescent development in smoking, drinking, and psychiatric distress were identified and support previous evidence of inter-relationships between smoking, drinking and psychiatric distress [3–5]. A *Low Risk* class had low levels of smoking and drinking, and low but increasing levels of psychiatric symptoms. Compared with this group, smokers had raised risks for drinking and psychiatric distress, and the majority of smokers were in the *Early Smokers* class where drinking and distress tended to develop after smoking initiation. This supports previous research showing prospective relationships between adolescent smoking and later problematic alcohol use and mental health problems [5]. On the other hand, patterns where drinking and distress developed without smoking were also relatively common.

The findings were contrary to what would be expected if SEP were a simple, common cause of these outcomes; the *Early Smokers* were the only class for which a disadvantaged SEP was associated with a higher likelihood of membership. In the *High Drinking* and *Late Smokers* classes, which both included increased risks for drinking and distress, there was either no association with SEP or an association in the opposite direction. For the *High Distress* class associations with SEP were inconsistent, most showed no effect but some measures showed associations in opposite directions, and thus this probably represents the more specific characteristics of each SEP measure more than SEP in general, suggesting a weak relationship with SEP. Adolescents in more deprived areas stood out as unlikely to be in the *Late Smokers* and *High Distress* classes. Both of these classes had high levels of distress, suggesting there may be something particular about more deprived areas (e.g., solidarity, social cohesion) that is protective in terms of distress. On the other hand, this may represent a cultural bias against reporting such symptoms within more deprived areas.

As smoking in the *Early Smokers* class tended to precede problems with drinking and distress, it may be that a disadvantaged SEP promotes early uptake of smoking only, and this then acts as a causal factor leading to later problems with drinking and psychiatric distress [5]. This could mean that the obvious benefits of preventing early smoking uptake among disadvantaged adolescents would additionally include beneficial effects on inequalities in distress and drinking. Alternatively, early smoking might not be causal but may instead be a marker for individual psychiatric vulnerability or for particular experiences within a disadvantaged SEP, either of which could then also lead to drinking problems and psychiatric symptoms. Indeed, the findings may represent an interaction between SEP and vulnerability for substance use and distress. Vulnerability in a disadvantaged SEP could lead to the *Early Smoking* developmental pattern described, while vulnerability in a more advantaged SEP leads into the *High Drinking* pattern.

Inconsistent associations between drinking and SEP have previously led some to suggest that two opposing processes link SEP and drinking; that is, a lower SEP is generally associated with poorer health including heavier drinking, while a higher SEP indicates more resources for obtaining alcohol [33]. These opposing processes could also be linked to different motivations for drinking; while some use alcohol to enhance pleasure, others use it as a mechanism for coping with stress [8,34]. The adverse stressors and lack of other coping resources associated with socioeconomic disadvantage could promote coping-motivated drinking, while those of higher SEP have more resources to enable drinking for pleasure. Given that smokers often view smoking as a coping mechanism for dealing with stress [11], smoking that begins early and is maintained at increasingly heavier levels across late adolescence, as seen in the *Early Smokers* class, may be a marker for stress-related processes within a disadvantaged SEP, which may then also promote coping-motivated drinking. If drinking in the *High Drinking* class represented more pleasure-motivated drinking then this might explain why this pattern was somewhat more likely for those in a more affluent SEP. Alternatively, there may be other processes of socioeconomic disadvantage that promote both early smoking and drinking, such as fewer alternative activities or lower quality parental monitoring [35,36].

Opposing processes might also explain why previous research from the Twenty-07 Study has indicated late adolescence as a period of relative equality in psychiatric distress [37,38]. Adolescents in more affluent areas, for example, may experience anxiety-promoting pressure to do well in education [20], while adolescents in disadvantaged circumstances experience other kinds of stress or lower levels of coping resources, leading both to increased psychiatric symptoms and other problems such as early smoking. If adolescent distress in an affluent SEP is associated mainly with education and tends to dissipate thereafter, while adolescent distress in a disadvantaged SEP is prompted by stressful life conditions that persist into adulthood, this may create socioeconomic inequalities in distress that widen with age [38].

These findings are presented with some caveats. The drinking measurements combined quantity and frequency, which might not have adequately reflected the consumption of those who drank heavily but infrequently, though previous research suggests that only a minority of adolescents drink this way [34]. Similarly, the smoking measurements may not have captured heavy smoking that occurred infrequently (i.e., less than weekly). If drop-out was associated with particular response patterns then the prevalence of these patterns may have been somewhat underestimated. With respect to SEP, however, the clearest effects were in relation to the *Early Smokers* class, many of whom would have been identifiable from the baseline data due to their early smoking. Thus the small differences in drop-out by SEP are unlikely to have greatly influenced the results. Also, the data refer to the specific geographic and temporal context of the West of Scotland in the late 1980s and early 1990s. Different developmental patterns and associations with SEP might be evident in other contexts where outcomes are more or less prevalent. For example, more recent female cohorts from this region have higher prevalence rates for all outcomes [20,21]. Nevertheless, studies of developmental trajectories for individual outcomes in other contexts have identified broadly similar trajectories to those evident here. For example, U.S. studies have, for the ages studied here, distinguished between early and late onset smoking [39], between light drinking and increasingly heavy drinking [34], and among very high, consistently low, or moderate but increasing levels of depressive symptoms [40]. Our findings replicate most of these patterns, but also indicate how they co-occur, and how SEP is associated with particular combinations of trajectories.

Examining adolescent development across all three outcomes—smoking, drinking, and psychiatric distress—suggests opposing processes linking drinking and distress to SEP contingent upon early smoking. Such opposing processes could be missed in research that focuses on only one outcome at a time, as the opposition would result in weak or null associations. A key area for further research seems to be in determining whether early smoking makes a causal contribution to later drinking and distress, or is merely a marker for other causal processes related to a disadvantaged SEP. If early smoking is causal, then intervening to prevent smoking in early adolescence may be especially important, whereas if it is a marker for other processes it is important to understand what those processes are so that appropriate interventions can be devised.

## Figures and Tables

**Table A1:** Model fit statistics for determining number of latent classes

Number of Classes	Log likelihood	AIC^a^	BIC^b^	% of Residuals^c^ >4	Entropy	Identification^d^
2	−11,763.78	23,637.56	23,930.26	12	.868	100
3	−11,650.07	23,466.14	23,907.85	10	.708	100
4	−11,559.13	23,340.26	23,930.98	7	.750	100
5	−11,502.42	23,282.84	24,022.58	5	.725	65
6	−11,449.76	23,233.52	24,122.27	3	.743	15
7	−11,413.17	23,216.35	24,254.11	2	.735	5

a AIC = Akaike's Information Criterion.

b BIC = Bayesian Information Criterion.

c 2-way item-by-item standardized residuals.

d Identification represents the % of times the best-fitting solution was replicated out of 20 sets of starting values. These 20 sets of starting values were identified by following 250 sets of starting values for 20 iterations and selecting those with the best log likelihood values.

**Table A2:** Odds ratios for latent class membership using modal assignment^a^

	Latent Class (ref: Low Risk)											
	High drinking		*p*	Early smokers		*p*	Late smokers		*p*	High distress		*p*
	OR	95% CI		OR	95% CI		OR	95% CI		OR	95% CI	
Males	1	–		1	–		1	–		1	–	
Females	.68	.51–.92	.012	.83	.63–1.10	.193	1.50	.99–2.28	.055	1.99	1.30–3.05	.002
Nonmanual household	1	–		1	–		1	–		1	–	
Manual household	.73	.53–1.00	.053	1.85	1.40–2.44	<.001	.98	.64–1.49	.915	1.04	.69–1.58	.834
Couple parents	1	–		1	–		1	–		1	–	
Single parents	1.19	.76–1.88	.444	1.96	1.34–2.87	.001	.99	.52–1.91	.986	1.74	1.01–3.00	.047
Owned home/mortgage	1	–		1	–		1	–		1	–	
Rented/other home	.62	.46–.83	.002	2.23	1.65–3.01	<.001	.92	.61–1.39	.697	.86	.58–1.30	.480
Parent(s) in school after age 16 years	1	–		1	–		1	–		1	–	
Parent(s) left school by age 16 years	.79	.58–1.08	.142	1.86	1.36–2.54	<.001	.82	.54–1.25	.353	.63	.42–.95	.029
Parent(s) in full-time employment	1	–		1	–		1	–		1	–	
Parent(s) in part-time employment	1.06	.62–1.82	.832	1.70	1.06–2.73	.027	.78	.34–1.79	.562	.88	.38–2.02	.756
Parent(s) not employed	.70	.46–1.06	.093	1.75	1.26–2.44	.001	.74	.42–1.31	.302	1.45	.90–2.33	.130
Top income tertile	1	–		1	–		1	–		1	–	
Middle income tertile	.77	.54–1.10	.157	1.59	1.11–2.27	.012	.79	.48–1.28	.335	.67	.40–1.11	.121
Bottom income tertile	.68	.47–.99	.044	2.26	1.59–3.22	<.001	.88	.54–1.45	.627	1.02	.63–1.65	.930
Least deprived areas	1	–		1	–		1	–		1	–	
Middling area deprivation	.87	.57–1.33	.517	1.15	.72–1.82	.565	.42	.25–.70	.001	.52	.30–.92	.024
Most deprived areas	.49	.31–.77	.002	1.45	.92–2.29	.110	.32	.19–.56	<.001	.54	.31–.95	.032

a All ORs are adjusted for gender except those for gender which are unadjusted.

**Table 1:** Descriptive statistics for baseline covariates and attrition^a^

	Baseline interview: Age 15		Postal follow-up: Age 17		Follow-up interview: Age 18	
N (%)	1,515	(100)	1,250	(82.5)	1,343	(88.6)
	N	%	N	%	N	%
Baseline characteristics						
Gender						
Male	737	48.6	581	46.5	638	47.5
Female	778	51.4	669	53.5	705	52.5
Household social class						
Nonmanual	891	59.8	769	62.3	827	62.4
Manual	598	40.2	465	37.7	498	37.6
Lone parenthood						
Couple parents	1,273	86.3	1,077	88.1	1,143	87.1
Single parent	202	13.7	145	11.9	170	12.9
Housing tenure						
owned	641	43.1	574	46.6	607	45.8
rented	847	56.9	658	53.4	717	54.2
Parental education						
Post-16	519	34.9	458	37.2	489	37.0
Left by 16	969	65.1	774	62.8	834	63.0
Parental employment status						
Full-time	1,059	71.2	911	74.1	975	73.7
Part-time	124	8.3	97	7.9	113	8.5
Not employed	304	20.4	221	18.0	235	17.8
Household income						
Top tertile	471	33.3	425	36.2	450	35.6
Mid-tertile	473	33.4	389	33.1	427	33.8
Bottom tertile	472	33.3	361	30.7	388	30.7
Area deprivation						
Least deprived	242	16.0	221	17.7	233	17.4
Middling	648	42.8	550	44.0	592	44.1
Most deprived	624	41.2	478	38.3	517	38.5

a Summary statistics are based on valid responses. Item-missingness was generally lower than 5% except for baseline household income (6.4%, 6%, and 5.8% at ages 15, 17, and 18 years).

**Table 2:** Frequency of smoking, drinking and psychiatric distress at each measurement point^a^

	Baseline interview: Age 15		Postal follow-up: Age 17		Follow-up interview: Age 18	
N (%)	1,515	(100)	1,250	(82.5)	1,343	(88.6)
	N	%	N	%	N	%
Outcomes						
Smoking^b^						
None	1,225	81.3	895	72.1	881	65.8
Low	48	3.2	32	2.6	24	1.8
Medium	170	11.3	172	13.9	118	8.8
High	64	4.2	142	11.4	315	23.5
Drinking^c^						
None	174	11.5	212	17.0	123	9.9
Low	1,040	68.9	704	56.5	361	29.0
Medium	210	13.9	274	22.0	497	40.0
High	86	5.7	55	4.4	262	21.1
Psychiatric distress^d^						
None	778	55.3	573	46.7	367	28.2
Low	415	29.5	315	25.7	399	30.7
Medium	132	9.4	165	13.4	319	24.5
High	83	5.9	174	14.2	216	16.6

a Summary statistics are based on valid responses. Missingness was generally lower than 5% except for psychiatric distress at baseline (7.1%) and drinking at age 18 (7.4%).

b Smoking: None, Light, Medium, and Heavy equate respectively to 0, <1, ≥1, and ≥10 cigarettes daily.

c Drinking: At baseline, None, Light, Medium, and Heavy equate respectively to no drinking, <monthly, ≥monthly, and ≥weekly. At the two follow-ups, None, Light, Medium, and Heavy equate respectively to no drinking, <weekly, ≥weekly and within limits (14/21 units), and ≥weekly and over limits (14/21 units).

d Psychiatric Distress: None, Light, Medium, and Heavy equate respectively to scores of 0, 1–2, 3–4, and 5 + on the GHQ-12.

**Table 3:** Odds ratios for latent class membership^a^

	Latent class (ref: low risk)											
	High drinking		*p*	Early smokers		*p*	Late smokers		*p*	High distress		*p*
	OR	95% CI		OR	95% CI		OR	95% CI		OR	95% CI	
Males	1	–		1	–		1	–		1	–	
Females	.43	.23–.81	.008	.78	.58–1.06	.113	2.04	1.02–4.10	.045	2.94	1.30–6.65	.009
Nonmanual household	1	–		1	–		1	–		1	–	
Manual household	.58	.30–1.11	.100	1.89	1.39–2.57	<.001	.84	.43–1.65	.606	.89	.44–1.80	.735
Couple parents	1	–		1	–		1	–		1	–	
Single parents	1.20	.52–2.78	.666	2.04	1.34–3.11	<.001	.87	.29–2.64	.807	2.31	1.08–4.95	.032
Owned home/mortgage	1	–		1	–		1	–		1	–	
Rented/other home	.41	.23–.75	.003	2.38	1.69–3.34	<.001	.76	.41–1.41	.385	.92	.48–1.73	.786
Parent(s) in school after age 16 years	1	–		1	–		1	–		1	–	
Parent(s) left school by age 16 years	.71	.40–1.27	.251	2.04	1.43–2.92	<.001	.63	.34–1.15	.130	.57	.30–1.08	.086
Parent(s) in full-time employment	1	–		1	–		1	–		1	–	
Parent(s) in part-time employment	1.23	.51–2.97	.648	1.91	1.14–3.20	.014	.50	.09–2.87	.437	1.16	.33–4.07	.815
Parent(s) not employed	.45	.16–1.26	.131	1.83	1.28–2.62	.001	.48	.16–1.47	.199	1.80	.89–3.62	.101
Top income tertile	1	–		1	–		1	–		1	–	
Middle income tertile	.64	.34–1.22	.174	1.65	1.10–2.49	.016	.65	.32–1.32	.236	.57	.24–1.32	.186
Bottom income tertile	.50	.24–1.05	.066	2.42	1.62–3.61	<.001	.65	.30–1.41	.274	1.01	.49–2.08	.980
Least deprived areas	1	–		1	–		1	–		1	–	
Middling area deprivation	.93	.43–2.02	.859	1.18	.68–2.04	.561	.31	.15–.61	.001	.27	.11–.66	.004
Most deprived areas	.29	.10–.80	.017	1.51	.88–2.59	.137	.19	.08–.43	<.001	.38	.17–.83	.015

a All ORs are adjusted for gender except those for gender, which are unadjusted.

## Appendix 1 Determining the Number of Latent Classes

The number of latent classes was determined by estimating a series of latent class models each with an incrementally greater number of latent classes. These models were then compared on the basis of various model-fit statistics, aiming for an optimal balance of fit and parsimony, that is, the lowest number of classes that could adequately describe the data [1]. The log likelihood, Akaike's Information Criterion (AIC) [2] and Bayesian Information Criterion (BIC) [3] are all measures of how well the model fits the observed data. Higher values for the log likelihood and lower values for the AIC and BIC indicate better fit. The AIC and BIC also both take into account the parsimony of the model (i.e., the number of model parameters being estimated), with the BIC being more stringent in terms of parsimony, taking the sample size into account as well as the number of parameters. Standardized bivariate marginal residuals can also be used to assess model fit with values greater than four representing poor fit [4,5]. Here the percentage of all standardized bivariate residuals exceeding four is employed as a summary statistic [5]. As a rule of thumb, fit is considered to be adequate if less than 10% of these residuals exceed 4. Entropy indicates how definitively respondents are being classified into latent classes (values range from 0 to 1 with 1 representing definitive classification) [6]. Because, for example, a 1-class model would perfectly classify respondents, but probably have poor fit, entropy is only really considered in situations where models do not differ much in terms of fit (in which circumstances a more definitive classification is preferred). Identification is the percentage of different sets of starting values that produced the best-fitting model and gives an indication of whether one can be confident that the model represents a global rather than a local maximization of model fit. If 100% of the sets of starting values converge to the same solution, then one can be confident that this represents a global maximum. Where the best-fitting solution is hard to replicate (i.e., it is reproduced in only a small percentage of the sets of starting values) it may well represent a local maximum or the model may not be identified [7].

Because these various model fit statistics often disagree, it is possible that more than one model would appear as a viable candidate for an optimal summary of the data, in which case additional criteria relating to the interpretive value of the latent classes were employed. More parsimonious models, that is, with fewer classes, were preferred *a priori*. The interpretive value of additional classes was considered to be related to their prevalence and the uniqueness of the response probability profile. Additional classes that only represented a very small proportion of the sample, or which did not have very different response probability profiles from other classes, would not have added much interpretive value and could be accepted as noise within a more restricted classification. In contrast, additional classes that represented a sizeable proportion of the sample and had very distinct response probability profiles would ideally have been included for their extra interpretive value.

Table A1 shows the model fit statistics for latent class models with two through seven latent classes. Models with additional classes were not considered because it was becoming difficult to replicate the best-fitting solutions (meaning that they could represent local maxima). The BIC had its lowest value at three classes, but values for the log likelihood, AIC, and standardized bivariate marginal residuals continued to improve with higher numbers of classes. The standardized bivariate marginal residuals were only at the threshold of acceptability for the three-class model. Entropy statistics also indicated a preference for higher numbers of classes over the three-class model. Thus it was not immediately clear which model should be considered optimal as some indicators pointed toward a three-class model while others pointed toward models with additional classes.

In order to resolve this ambiguity the response probability profiles were inspected, starting with the three-class model, and then comparing models with additional classes, until they no longer suggested a meaningful addition to the model. This process led to the selection of the five-class model as the optimal representation of the data. The three-class model indicated patterns similar to the *Low Risk*, *High Drinking*, and *Early Smokers* classes presented in the paper (i.e., the three most prevalent), but there was less differentiation between these classes in terms of psychiatric distress. A four-class model drew out the *High Distress* pattern, which seemed to be an informative addition, considering that it also resulted in greater differentiation between the distress levels of the other classes. The five-class model differentiated between the *Early Smokers* and *Late Smokers*, which seemed to be a theoretically valuable distinction. The six-class model identified a small subgroup (approximately 6% of the sample) within the *Low Risk* class, who did not really start drinking at all until age 18. Because this group was relatively small and was only clearly differentiated from the *Low Risk* class in terms of no versus light drinking at younger ages, this was not considered a valuable addition and the five-class model was chosen.

## Appendix 2 Odds Ratios for Modal Assignment Method

Table A2 shows the Odds Ratios (ORs) for latent class membership based on modal assignment of respondents into classes. The results are similar to those presented using the Vermunt 3-step method [8,9] except that the magnitude of associations is somewhat smaller and the confidence intervals somewhat narrower.
